# Serum Level of Lactate Dehydrogenase is a Useful Clinical Marker to Monitor Progressive Multiple Myeloma Diseases: A Case Report

**DOI:** 10.4274/Tjh.2013.0044

**Published:** 2014-03-05

**Authors:** Hava Üsküdar Teke, Mustafa Başak, Deniz Teke, Mehmet Kanbay

**Affiliations:** 1 Kayseri Education and Research Hospital, Department of Hematology, Kayseri, Turkey; 2 Kayseri Education and Research Hospital, Department of Internal Medicine, Kayseri, Turkey; 3 Kayseri Education and Research Hospital, Department of Cardiology, Kayseri, Turkey; 4 Kayseri Education and Research Hospital, Department of Nephrology, Kayseri, Turkey

**Keywords:** Multiple myeloma, LDH, prognosis

## Abstract

To follow the progression of multiple myeloma (MM) disease, serum lactate dehydrogenase (LDH) levels are as useful markers as beta-2 microglobulin and monoclonal immunoglobulin. With this study, we have presented a case of a patient with a multiple myeloma which was fulminant course, whose LDH levels were normal at the onset of diagnosis increasing as 27 times more than normal as the disease progressed and who showed the development of extramedullary plasmacytomas. The patient, an 80-year-old female, was diagnosed with stage IIIA IgA type multiple myeloma and melphalan-prednisolon (MP) treatment was started. Although the LDH levels were low during the diagnosis and chemotherapy, the LDH levels increased up to 7557 U/L following the progression and occurrence of extramedullary plasmacytomas and the patient died. During the observation of the patient with MM, if the LDH levels are abnormally high, the progression of the disease should be considered after eliminating the other causes. Bone marrow aspiration and biopsy should be examined and the progression or relapse should be shown. On the other hand, the patients with LDH levels are high should be considered to have added plasmacytomas, the whole body should be examined at an early stage before the development of clinical symptoms and early treatment should be started.

## Abstract (tr)

Serum laktat dehidrogenaz (LDH) düzeyleri, multiple myeloma (MM) hastalık ativitesinin takibi için beta-2 mikroglobulin ve monoklonal immunglobulin kadar kullanışlı bir markırdır. Bu yazıda tanı aşamasında LDH değerleri normal iken, takibinde LDH seviyeleri normalin 27 katı kadar artan ve ekstramedüller plazmasitom gelişen, fulminan seyirli bir multiple myeloma olgusunu sunduk. Seksen yaşındaki bayan hasta, Evre3A IgA tipi multiple myeloma tanısı konularak MP (melphalan-prednizolon) tedavisi başlanan hasta, tanı dönemi ve kemoterapi süresince LDH düzeyleri düşük seyretmesine rağmen progresyon sonrası ve kliniğe eklenen ekstramedüller plazmasitomlar ile LDH düzeyleri 7557 U/L düzeyine kadar çıkmış ve hasta fatal seyretmiştir. MM’li hastaların takibi sırasında LDH değerlerinde olan anormal yüksekliklerde diğer nedenler ekarte edildikten sonra hastalık progresyonu düşünülmelidir. Kemik iliği ve laboratuvar değerlendirmeleri ile progresyon veya nüks gösterilmelidir. LDH seviyeleri yüksek olan hastalarda ise mutlaka ilave olmuş plazmasitomların varlığı düşünülmeli, klinik bulgular gelişmeden erken dönemde tüm vücut taraması yapılmalı ve erken tedavi başlanmalıdır.

## INTRODUCTION

Multiple myeloma is a malignant hematological disease which is characterized by monoclonal immunoglobulin production and malignant proliferation of plasma cells in bone marrow. Serum beta2-microglobulin, serum albumin, platelet count, serum creatinine and age are important markers for survival during the course of disease [1]. Moreover; among these prognostic markers, elevated LDH is correlated with beta2-microglobulin [2]. Elevated LDH levels are observed rarely at the onset of the multiple myeloma; however, as the disease progresses LDH levels increase to levels higher than those at diagnosis [3]. The median overall survival of the patients whose LDH levels are high is shorter than those patients whose LDH levels are normal [4]. As LDH gives an idea about the level of tumor mass, the increase of LDH during the course of the disease may refer to the increased levels of tumor, relapse or the existence of extra plasmacytomas [5].

With this study, we have presented a case of a patient with a multiple myeloma which was fulminant course, whose LDH levels were normal at the onset of diagnosis increasing as 27 times more than normal as the disease progressed and who showed the development of extramedullary plasmacytomas. 

## CASE REPORT

**Figure 1:**
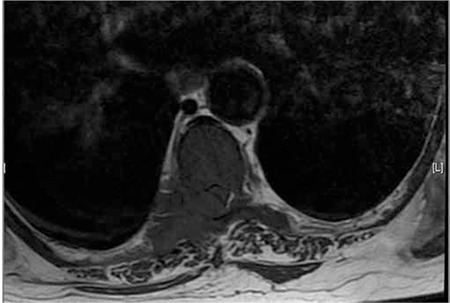
The patient plasmocytomas in the Lomber MR

**Figure 2:**
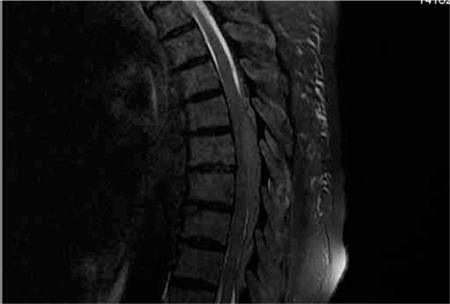
Plasmocytomas in the lomber, thorakal area in MR.

An 80 years-old female patient. The patient, who was led to hematology policlinic following the findings of anemia (hemoglobin 8.16 gr/dl) and leucopenia (White blood cell 2.9x103/uL) with the complaints of fatigue in May 2011, had no any other disease except from hypertension. As the patient who was examined for bicytopenia had higher level of Ig A and suspicious monoclonal peak was observed in serum protein electrophoresis was evaluated. Immunofixation electrophoresis results have shown ‘IgA/k’ monoclonal band with IgA levels of 2610 mg/dl and kappa levels of 2040 mg/dl. After the patient was examined for bone marrow aspiration-biopsy and flow cytometry, in the bone marrow biopsy the patient was found to have CD38, CD138 with the level 30%, and kappa positive plasma cells. Direct radiographies did not show any lytic lesions. The levels of C-reactive protein (CRP) and LDH were normal, beta-2 microglobulin level was 8.5 mg/L. The patient was diagnosed with stage IIIA IgA type multiple myeloma and MP treatment was started. The patient’s levels of IgA and monoclonality who was treated with five cures MP decreased during the treatment. Levels of LDH did not increase. The patient was not treated with 6th cure chemotherapy because of the infection. Thorax tomography did not show any pathology related to infection. Blood culture results were negative, E. coli has grown in urine culture. The patient’s LDH levels, whose fever decreased with the treatment of antibiotics, increased. Hemolysis tests which were done because of the high LDH levels (1808 U/L) were normal and fragmentation was not observed in peripheral blood smear. Ultrasonography results have showed that liver, pancreas and spleen were normal. As the patient had thrombocytopenia and bone pain, bone marrow aspiration-biopsy was examined to confirm the presence of resistance multiple myeloma. Bone marrow biopsy results have showed that plasma cells with kappa monoclonality have increased to the levels of 50%. The patient’s cerebral magnetic resonance (MR), who showed sudden loss of mass strength around the right lower extremity, was normal. Lumbar MR has showed that there has been a soft tissue mass around sacral 3-4 vertebra anterior paravertebral field and increasing numbers of tissue around the L1-L2 vertebra corpus anterior field were observed. Thoracal MR has showed that there has been soft tissue mass around T 5-9 vertebra which causes invasion in posterior units (Figure 1, Figure 2). Informed consent was obtained.

Our patient’s laboratory results were given in the Graphic 1.

The patient was taken for operation by neurosurgery. Following the pathology results, the patient was diagnosed with plasmacytoma. The patient was treated with weekly dexamethasone. During the dexamethasone treatment the LDH levels decreased from 7557 U/L to 2000 U/L. Radiotherapy treatment was planned for the patient; however, sudden onset of dyspnea and loss of consciousness were observed with the patient. The cerebral CT results did not show any sign of hemorrhage and emboli, edema was observed. The patient, who had been observed to have diffuse lung edema following the X-ray, was put under dialysis. Afterwards, the patient died of lung edema during the dialysis. 

## DISCUSSION

Symptoms of multiple myeloma which are caused by plasma cells in bone marrow are heterogeneous and related to tumor mass. While the most common symptom of MM is bone pain, the most common laboratory result is anemia. In our case, also, anemia was observed which is the most common symptom of MM; however, laboratory results showed leukopenia which is rarely seen. Being associated with prognosis, the levels of beta2-microglobulin, plasma cell labeling index, CRP and IL-6 are parameters with MM patients. High levels of LDH are associated with advanced disease and poor survival. At the onset of MM, high levels of LDH are seen rarely; however, as the disease advanced, the levels of LDH increase to levels higher than those at diagnosis [3]. Also in our case; at the onset of the disease, the levels of LDH were normal and the levels of LDH increased as the disease progressed. According to the research; LDH, international staging systems, performance status, age and platelet counts were shown as independent prognostic factors. The median overall survival of the patients with high and normal LDH was 15 vs. 44 months [6]. While the overall survival of the patients with MM whose LDH levels were above 250 U/L was 4 months that of the patients with lower LDH levels was 20 months [7]. Although in our case the LDH levels were low during the diagnosis and chemotherapy, the LDH levels increased to 7557 U/L following the progression and occurrence of extramedullary plasmacytomas and the patient died. LDH is a cytoplasmic enzyme and may have been observed in nearly all major organ cells. If cells lysis occurs, or cells and membranes are damaged, cytoplasmic enzymes, such as LDH are released into the extracellular area [8]. LDH isoenzymes are present in brain, kidney, liver, lung, lymph nodes, myocardium, skeletal muscle, spleen, erythrocytes, leucocytes and also platelets and divided into 5 components [9]. LDH-3 isoenzyme is especially present in lung diseases and special tumors; LDH-4 is observed in kidney, placenta and pancreas and especially can be found high in pancreatitis. Researches have shown that total LDH, LDH-2, LDH-3 and LDH-4 isoenzymes are high with untreated leukemia patients [10]. In our case, total LDH level was found as 7557 U/L which has never occurred in the literature. Other clinical situations such as hemolytic anemia, pneumonia, pancreatitis which cause the high levels of LDH have been eliminated. LDH-3 and LDH-4 isoenzymes are found at high levels in our case. According to these results, it may be concluded that prognosis will be poor, when the total LDH, LDH-3 and LDH-4 isoenzymes are high with the patients of MM. New studies about LDH isoenzymes of the patients with MM, whose LDH levels are high, are required to confirm this hypothesis. 

 To conclude, to follow the progression of MM disease, serum LDH levels are as useful markers as beta-2 microglobulin and monoclonal immunoglobulin. During the observation of the patient with MM, if the LDH levels are abnormally high, the progression of the disease should be considered after eliminating the other causes. Bone marrow aspiration and biopsy should be examined and the progression or relapse should be shown. On the other hand, the patients with high levels of LDH should be considered to have added plasmacytomas, the whole body should be examined at an early stage before the development of clinical symptoms and early treatment should be started.

## CONFLICT OF INTEREST STATEMENT

The authors of this paper have no conflicts of interest, including specific financial interests, relationships, and/ or affiliations relevant to the subject matter or materials included.

## Figures and Tables

**Figure 3:**
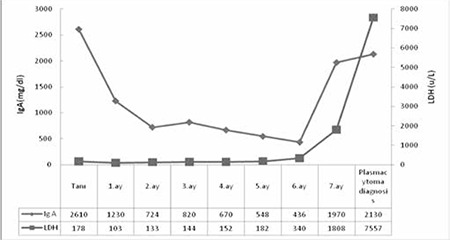
Our patient’s laboratory results
